# 2D Representation of Transcriptomes by t-SNE Exposes Relatedness between Human Tissues

**DOI:** 10.1371/journal.pone.0149853

**Published:** 2016-02-23

**Authors:** Erdogan Taskesen, Marcel J. T. Reinders

**Affiliations:** Delft Bioinformatics Lab (DBL), Delft University of Technology, Delft, 2628CD, the Netherlands; University of Lausanne, SWITZERLAND

## Abstract

The GTEx Consortium reported that hierarchical clustering of RNA profiles from 25 unique tissue types among 1641 individuals accurately distinguished the tissue types, but a multidimensional scaling failed to generate a 2D projection of the data that separates tissue-subtypes. In this study we show that a projection by t-Distributed Stochastic Neighbor Embedding is in line with the cluster analysis which allows a more detailed examination and visualization of human tissue relationships.

## Introduction

The use of dimensionality reduction methods such as multidimensional scaling (MDS) or principal component analysis (PCA) are popular techniques for data representation in low dimensions[1]. As an example, the use of MDS was used in a recent study from the GTEx consortium[1] where transcriptome data of different human tissues[2] was projected in a 2D space. Their two dimensional map (2D-map) shows that blood samples are an outgroup which are in line with the hierarchical cluster (HC) analysis in the high dimensional representation[2, 3]. However, the majority of tissues grouped together in the 2D-map, which does not match their HC analysis[1]. A radial plot on seven metagenes[1] did show better separation of tissues compared to the MDS but still did not reflect the HC analysis fully.

When mapping high-dimensional genomic data to lower dimensions, it is important that distances between similar samples are preserved (instead of the overall distribution of samples). This allows separation of similar samples in the low dimensional representation, and hence discovery of relationships between nearby samples by visual inspection. We re-evaluated the projection of tissue types using the GTEx transcriptome data[1], consisting of RNA sequences of 52,576 unique gene transcripts and their abundance in each tissue (the gene RPKM values, as available in “RNA-SeQCv1.1.8 gene rpkm Pilot V3 patch1”). The use of t-Distributed Stochastic Neighbor Embedding (t-SNE) provides a 2D-map that accurately fits the general findings described previously[1–3]. In addition, we demonstrate that the generated 2D-map provides novel insights in the local relatedness between human tissues.

## Material and Methods

### Normalization

Normalization of the data is performed as described previously[1, 2]: genes were filtered with RPKM value > 0.1 in at least 80% of the samples, followed by a log2 transformation of the RPKM values (using a pseudocount of 1), and finally a zero-mean normalization. This resulted in gene expression profiles from a total of 16,142 protein-coding genes, pseudogenes, and long noncoding RNAs (lncRNAs). Note that 99.8% (16,115 out of 16,142) of the genes mapped to a single transcript.

### 2D mapping

**Fig 1:**
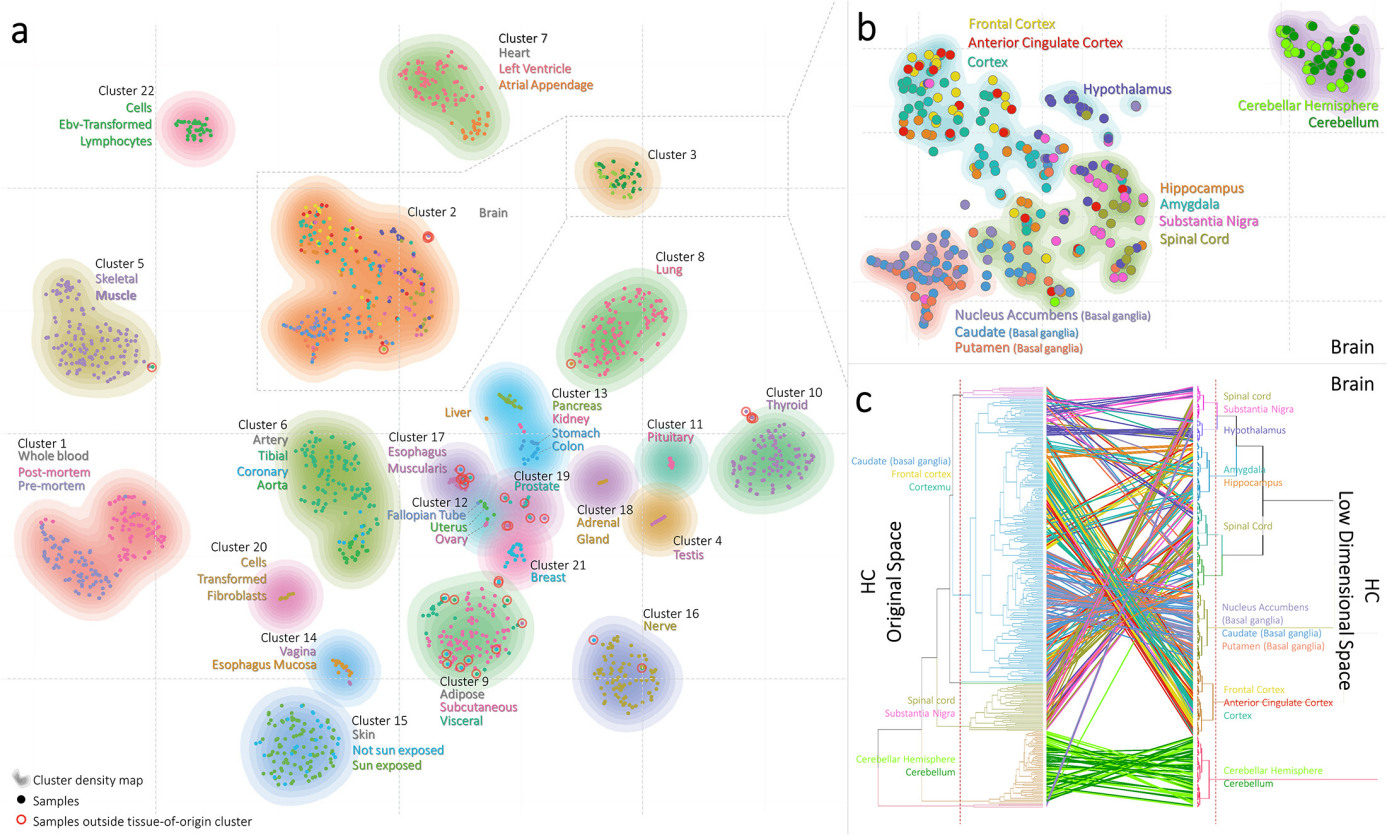
The tissue landscape of the gene expression profiles illustrated in a two-dimensional map. (A) Projection of 1641 GTEx samples in a 2D-map: Each point represents a sample which is coloured according to the (sub)tissue label (45 in total). Samples that cluster outside the matching tissue subtype-cluster are circled in red. Sample clusters are illustrated by the 22 differentially coloured density maps. (B) the projection of 313 brain samples and their associated brain-tissue. (C) Comparison of brain samples clustered by the HC approach versus the t-SNE approach resulted in cophenetic correlation[6] of 0.68. An edge links the sample ID positions between the HC and t-SNE clustering. Edge colours are based on the brain tissue regions. Clusters are labelled if a particular brain region was significantly overrepresented in the cluster (hypergeometric test with *P*≤0.001).

We utilized the Barnes-Hut t-SNE[4] algorithm to project the tissue samples in a 2D-map (t-SNE-map) using the gene expression profiles from 16,142 genes (Fig 1A). Barnes-Hut t-SNE non-linearly retains local similarities between samples at the cost of retaining the similarities between dissimilar samples. This is in contrast to methods such as PCA and MDS that use the same linear mapping to all data. As a result, t-SNE better preserves local (dis)similarities as they are not condensed due to the large dissimilarities in the data set. t-SNE learns this embedding by minimizing the Kullback–Leibler divergence between the probability distribution of the similarities between samples in the high dimensional space and the distribution of the similarities between samples in the 2D map, with respect to the positions of the samples in the 2D map (similarities are measured using Euclidean distances). The similarity of a sample to all other samples in the high dimensional space is modeled as Gaussian with the number of neighbors taken into consideration as a parameter (perplexity). For the low dimensional space this similarity is modelled as a Student t-distribution. The heavy-tail in the t-distribution ensures that distant samples do not condense the map, and as such the local similarities are better preserved. We ran Barnes-Hut t-SNE 1000 times and selected the solution with the lowest KL divergence. Note that Barnes-Hut t-SNE is an optimized version of t-SNE that can handle many samples with many features.

### Clustering

The tissue gene expression profiles in the 2D t-SNE map are clustered using DBSCAN[5]. Clustering cut-off (eps parameter) was chosen by maximizing the silhouette score. Samples were not forced into clusters but instead, 10% of the tissues samples (at most) can be labelled as ‘none-clustering’.

## Results

**Fig 2:**
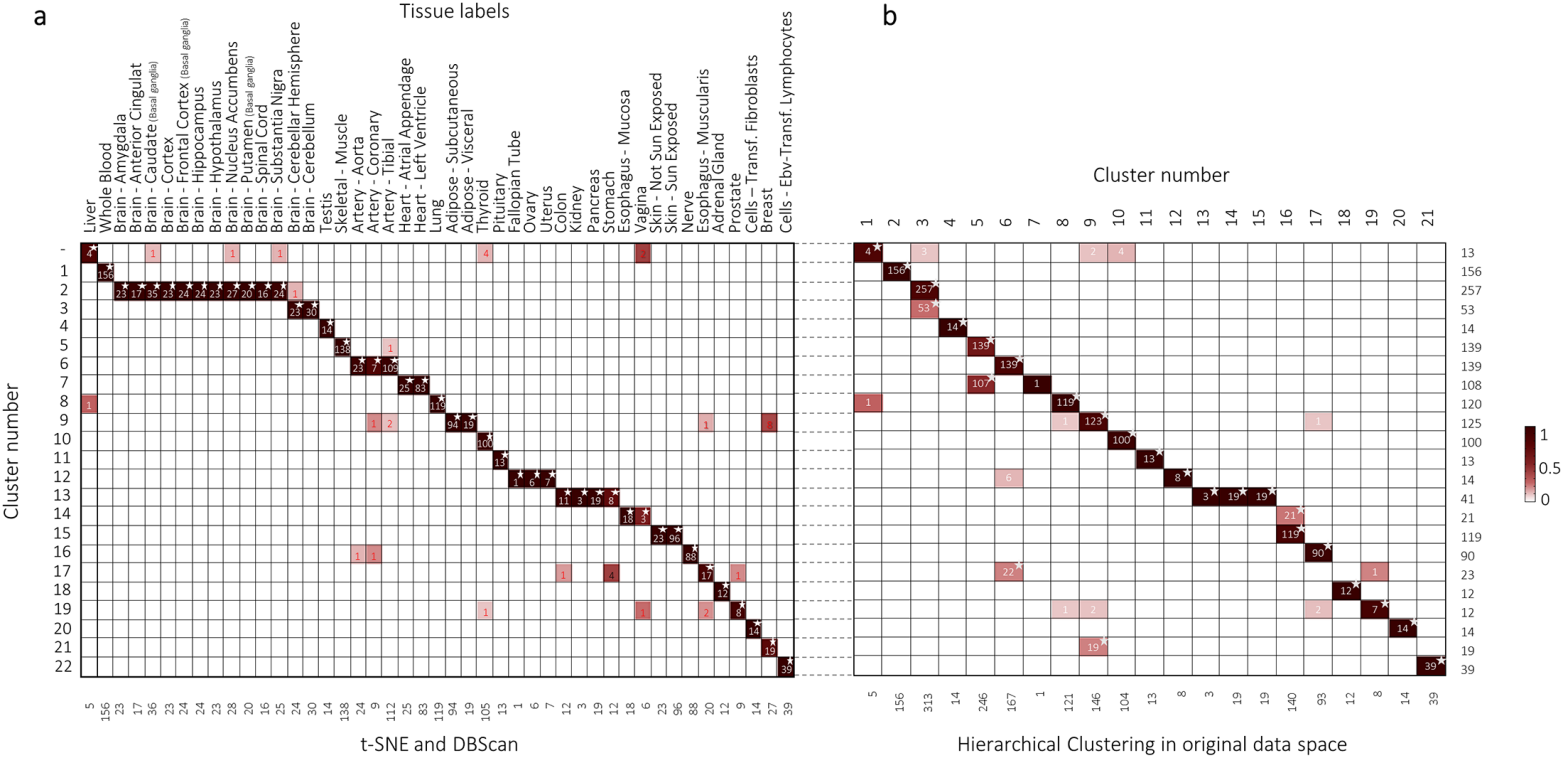
Cluster associations and comparison with the hierarchical clustering approach. (A) Association of the clusters based on the t-SNE-map with the tissue types. A star indicates significant overrepresentation (*P*≤0.0001) of samples in a cluster with the respective tissue label. Red-coloured numbers depict samples outside the cluster that matches their tissue label. Coloured squares depict the percentage of samples that overlap. (B) Comparison of the t-SNE-map based clusters versus the HC approach[2]. A star indicates significant over-representation (*P*≤0.0001) of samples in a cluster of the t-SNE-map with samples in a cluster of the HC approach[2]. Coloured squares depict the percentage of samples that overlap.

Based on our approach, the 25 unique tissues grouped into 22 clusters (Fig 2A). We characterized the clusters with a tissue label by means of the hypergeometric test with *P*≤0.0001 (Fig 2A). Out of the 22 detected clusters, 17 clusters showed near one-to-one relationships with the tissue label, two clusters contained major brain sub-regions; one cluster contained samples from the cerebellum and the other cluster contained 11 other brain sub-regions (Fig 1B), and three clusters showed a collection of multiple different tissues. The latter finding is not unexpected as these tissues represent respectively hormone-sensitive female reproductive tissues in cluster 12 (Fallopian tube, Ovary and Uterus samples), tissues associated with the digestive system in cluster 13 (Colon, Kidney, Pancreas and Stomach samples), and mucous-membrane tissues in cluster 14 (Esophagus and Vagina samples). Note that, although these tissues are grouped together in the cluster analysis, a visual look in the t-SNE map (Fig 1) demonstrates separation of the tissues. Furthermore, Besides the separation of tissues, the t-SNE-map also revealed substructures in the brain, blood and skeletal muscle tissues. For the 313 samples in the brain regions we did re-cluster using the initial t-SNE coordinates, and could demonstrate clear grouping of Cerebellar/Cerebellum regions, Basal Ganglia regions, Cortex regions, hypothalamus, and a mixture of Hippocampus, Amygdala, Substantia Nigra, and Spinal Cord regions were seen (Fig 1B). In whole blood we detected separation of pre- and post-mortem samples, and novel substructures were seen in skeletal muscle (Fig 1A) for which no further tissue annotation is available. Note that minimal differences in clustering results are observed if different gene expression level filtering cut-off values [0.5,…,10] are used (S1 and S2 Figs).

Twenty-one clusters overlapped significantly between the t-SNE-map and HC approach on the high-dimensional data (*P*≤0.0001) (Fig 2B). However, the HC approach grouped samples in larger clusters, e.g., all brain samples (HC cluster 2) or the skeletal muscle and heart tissue samples (HC cluster 5). Contrarily, the t-SNE-map clearly separates tissue types into different clusters (see also dendrogram differences in Fig 1C). A comparison between the HC in the original data space versus HC in low data space yielded in a cophenetic correlation[6] of 0.68, indicative of overlapping clusters.

To investigate the value of the t-SNE mapping further, we evaluated the results of different clustering algorithms (DBSCAN[5], HC, k-means, and Mixture of Gaussians) between the brain samples in the low dimensional t-SNE-map and in their original high dimensional representation. For all four clustering methods, the t-SNE-map resulted in higher significant cluster enrichment of the different brain regions (S1 Table), and lower Davies-Bouldin scores[7] (S3 Fig). This demonstrates that a reduction of data complexity, by a transformation step of samples into a low dimensional space, is beneficial for follow-up analysis. As an example, with the use of HC we detected eight clusters among the brain samples in both the original and low dimensional space but the clusters in the low dimensional map represented the different brain regions better (Fig 1C).

Interestingly, we also detected that the gene expression profiles of 31 samples that do not map to the cluster with a matching tissue label. These samples are either outlier tissues that are more heterogeneous at the cellular level[8] or may have been mislabelled (Fig 1A and S2 Table). This discrepancy was left unnoticed in the RNA-seq analysis[1–3], but the t-SNE-map clearly addresses the issue. Twelve out of 31 outliers lie in the adipose cluster 9 which is known to be a strong contaminant of other tissues samples as described in the pathology notes[2]. Out of the 12 samples, 8 samples originate from the neighbouring breast tissue cluster.

## Discussion

Taken together, we demonstrate that a 2D representation based on the t-SNE mapping of tissue-samples closely resembles the HC results and confirms the findings of the published GTEx studies. Our presented results are based on data that is derived from only the GTEx consortium. If multiple data sets from different consortiums are combined, it may require additional normalization steps or may even require batch-correction methods before making a joint analysis using t-SNE.

It should be noted that, besides the similarly clustered samples, differences between HC clustering in the original space versus the low dimensional space are also seen (Fig 1C). As an example, samples of the spinal cord are clustered in the original HC space (15 out of 16) whereas in the low dimensional space, these samples are divided into two clusters (Fig 1C). Interestingly, all samples (except for one) are in relative close proximity to each other in the 2D space (Fig 1B). Thus in terms of biological interpretation, there is one sample of the spinal cord which may be an outlier (clustered with Hypothalamus), and both approaches agree on that. In general, (small) differences between results are insurmountable when distinct approaches are used. A final biological interpretation remains an experts task for which our provided t-SNE-map allows a more detailed examination of local substructures across and within the human tissues.

## Supporting Information

S1 Fig Cluster associations for different pre-processing steps of the gene expressions. Cluster associations with the tissue labels are determined for 20 different gene filtering cut-offs, i.e., [0.5,…,10]. Each set of genes is reduced to 2 dimensions (by means of t-SNE) followed by DBSCAN cluster analysis to determine the optimal number of clusters. Each square depicts the -log10(P) enrichment of the cluster label with the tissue label, and shown when *P*≤0.001. Results are ordered on tissue label.
(TIF)

S2 Fig Summary of cluster associations with different pre-processing steps of the gene expression. Cluster associations with the tissue label are determined based on 20 different gene filtering cut-offs, i.e., [0.5,…,10], and are subsequently summarized. Per cut-off we determined the clustering, and each detected cluster is subsequently associated with the tissue labels (as demonstrated in S1 Fig). We now summarized the P-values for each of the 45 tissue labels by taking the minimum P-value over the clusters (e.g., minimum per column in S1 Fig). This results in a vector of P-values (one P-value for each tissue label) per cut-off. The 20 vectors are subsequently combined together for comparison.
(TIF)

S3 Fig Comparison of cluster algorithms in original space and low dimensional space using the brain samples. Four cluster algorithms are compared in the original data space and in the low dimensional space. Panel A-C depicts the Davies Bouldin index (DBindex) score for the clustering of brain samples in original data space using HC, Kmeans and Mixture of Gaussians respectively. DBSCAN was not able to fit the data in the original data space. Panel C-F depicts the DBindex score for the clustering of brain samples in the low dimensional space using HC, Kmeans, Mixture of Gaussians, and DBSCAN respectively. The red vertical line depicts the optimum number of clusters under the restriction that at least 5 and at most 15 clusters can exists. Panel G-J depicts the brain samples in the low dimensional space and the corresponding cluster labels for HC, Kmeans, Mixture of Gaussians and DBSCAN respectively.
(TIF)

S1 Table Comparison of four cluster algorithms in original data space and low dimensional space. Four cluster algorithms are compared in the original data space (using all available RNA-seq features) and in the low dimensional space (by first employing a t-SNE to two dimensions). Enrichment is computed for the detected cluster labels and the brain regions.
(TIFF)

S2 Table Samples that do not map to the cluster with a matching tissue label. Columns depict the tissue type. Rows depict the cluster number. Values within the cells depict the number of samples that do not map with a matching tissue label.
(TIFF)
