# Supplementary figures and images for: 2D Representation of Transcriptomes by t-SNE Exposes Relatedness between Human Tissues
Source: PLoS One. 2016 Feb 23;11(2):e0149853. doi: 10.1371/journal.pone.0149853 (PMC4764374; doi:10.1371/journal.pone.0149853)

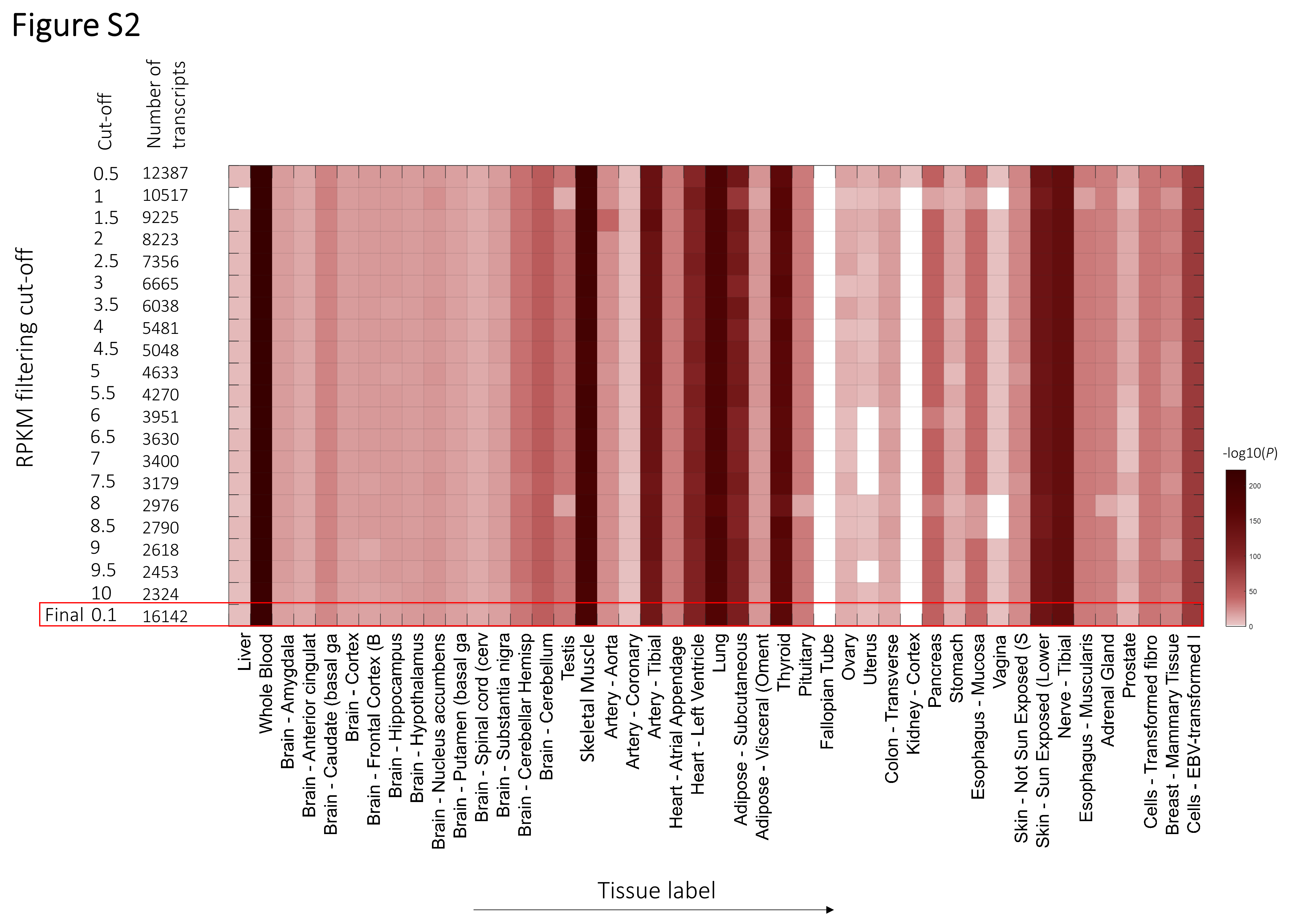

Supplement: S2 Fig — Cluster associations with the tissue label are determined based on 20 different gene filtering cut-offs, i.e., [0.5,…,10], and are subsequently summarized. Per cut-off we determined the clustering, and each detected cluster is subsequently associated with the tissue labels (as demonstrated in S1 Fig). We now summarized the P-values for each of the 45 tissue labels by taking the minimum P-value over the clusters (e.g., minimum per column in S1 Fig). This results in a vector of P-values (one P-value for each tissue label) per cut-off. The 20 vectors are subsequently combined together for comparison. (TIF) [file pone.0149853.s002.tif]

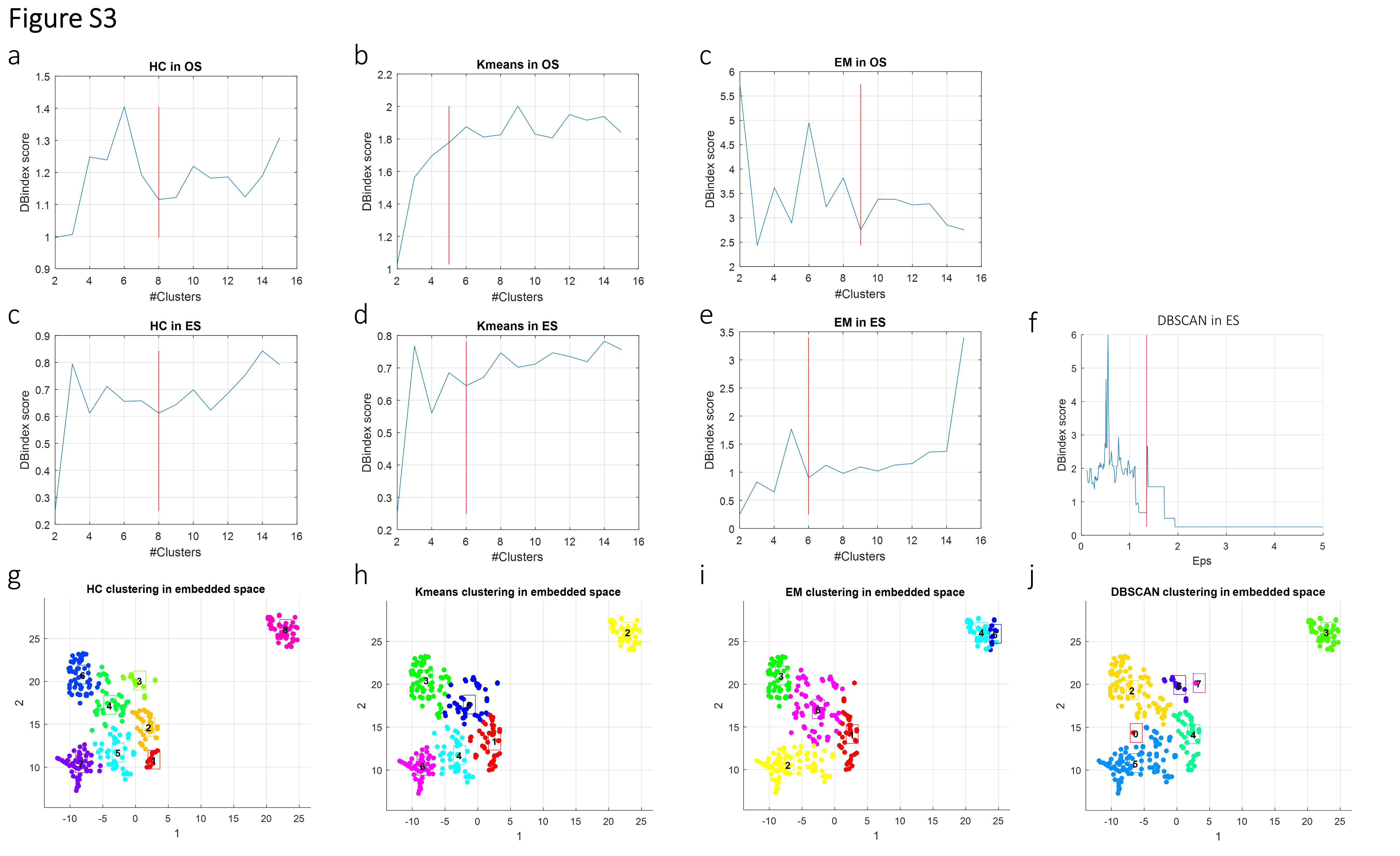

Supplement: S3 Fig — Four cluster algorithms are compared in the original data space and in the low dimensional space. Panel A-C depicts the Davies Bouldin index (DBindex) score for the clustering of brain samples in original data space using HC, Kmeans and Mixture of Gaussians respectively. DBSCAN was not able to fit the data in the original data space. Panel C-F depicts the DBindex score for the clustering of brain samples in the low dimensional space using HC, Kmeans, Mixture of Gaussians, and DBSCAN respectively. The red vertical line depicts the optimum number of clusters under the restriction that at least 5 and at most 15 clusters can exists. Panel G-J depicts the brain samples in the low dimensional space and the corresponding cluster labels for HC, Kmeans, Mixture of Gaussians and DBSCAN respectively. (TIF) [file pone.0149853.s003.tif]

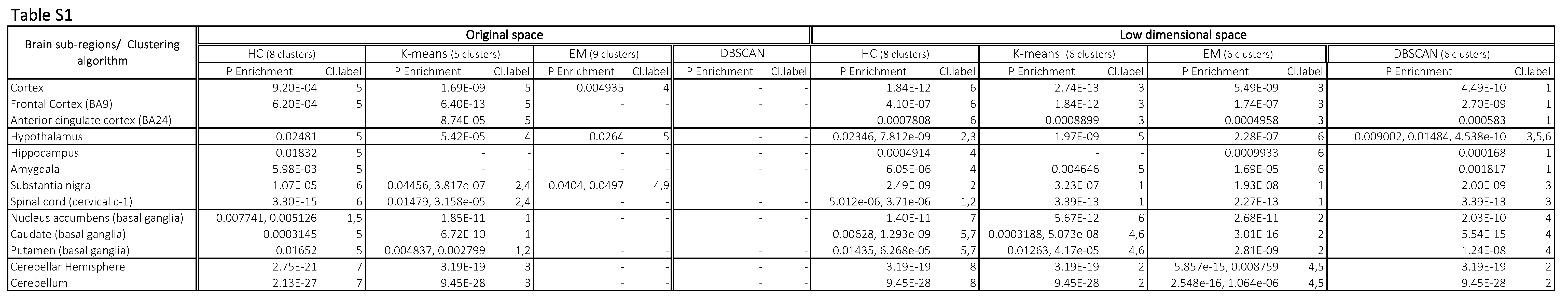

Supplement: S1 Table — Four cluster algorithms are compared in the original data space (using all available RNA-seq features) and in the low dimensional space (by first employing a t-SNE to two dimensions). Enrichment is computed for the detected cluster labels and the brain regions. (TIFF) [file pone.0149853.s004.tiff]

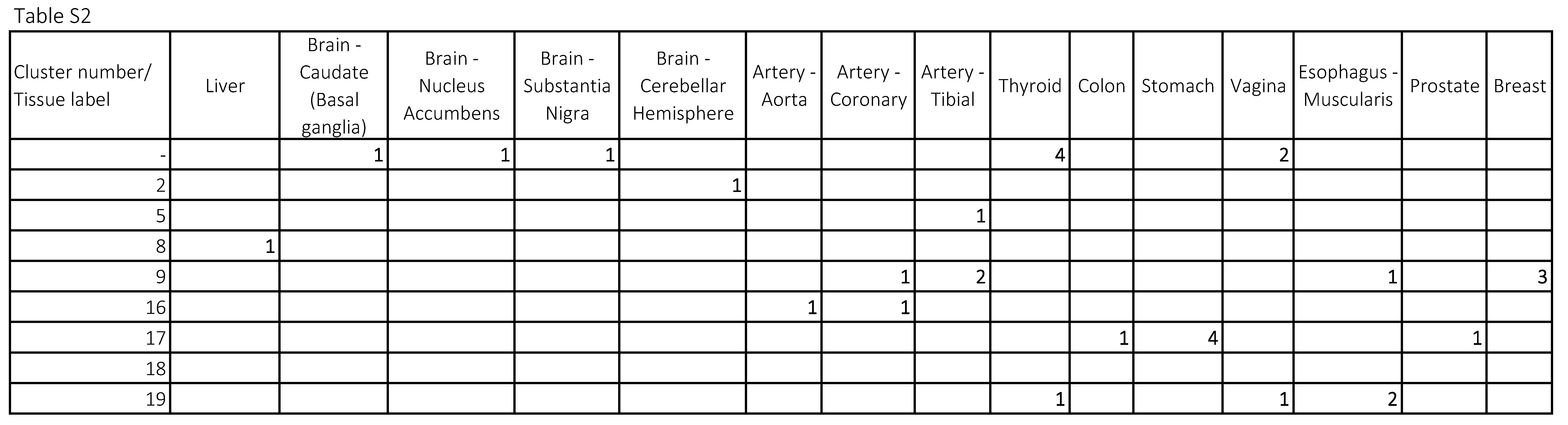

Supplement: S2 Table — Columns depict the tissue type. Rows depict the cluster number. Values within the cells depict the number of samples that do not map with a matching tissue label. (TIFF) [file pone.0149853.s005.tiff]
